# Darpp-32 and t-Darpp are differentially expressed in normal and malignant mouse mammary tissue

**DOI:** 10.1186/1476-4598-13-192

**Published:** 2014-08-15

**Authors:** Jessica L Christenson, Susan E Kane

**Affiliations:** Department of Cancer Biology, Beckman Research Institute at City of Hope, 1500 E. Duarte Road, Duarte, CA 91010 USA

**Keywords:** *PPP1R1B*, Darpp-32, t-Darpp, MMTV-Neu, MMTV-PyMT, Breast cancer, Tumorigenesis

## Abstract

**Background:**

Darpp-32 and t-Darpp are expressed in several forms of breast cancer. Both are transcribed from the gene *PPP1R1B* via alternative promoters. In humans, Darpp-32 is expressed in both normal and malignant breast tissue, whereas t-Darpp has only been found in malignant breast tissue. The exact biological functions of these proteins in the breast are not known. Although Darpp-32 is a well known regulator of neurotransmission, its role in other tissues and in cancer is less well understood. t-Darpp is known to increase cellular growth, inhibit apoptosis and contribute to acquired drug resistance. The use of transgenic mouse mammary tumor models to study Darpp-32 and t-Darpp in breast cancer *in vivo* has been limited by a lack of knowledge regarding t-Darpp expression in mice, in both normal and malignant tissue.

**Methods:**

We used RT-PCR and Western analysis to investigate Darpp-32 and t-Darpp levels in normal and malignant mouse mammary tissue. To determine if Darpp-32 and t-Darpp play a direct role in mammary tumor development, *Ppp1r1b* gene knockout mice and wild-type mice were crossed with a mouse mammary tumor model. Tumor growth and metastasis were examined. Differences between groups were determined by the two-tailed Student’s *t*-test.

**Results:**

We found that Darpp-32 was expressed in normal mouse mammary tissue and in some breast tumors, whereas t-Darpp was found exclusively in tumors, with t-Darpp usually expressed at equal or higher levels than Darpp-32. *Ppp1r1b* knockout in MMTV-PyMT transgenic tumor mice resulted in a decrease in tumor growth.

**Conclusions:**

The shift in expression from Darpp-32 to t-Darpp during mouse mammary tumorigenesis is reminiscent of the expression patterns observed in humans and is consistent with a role for t-Darpp in promoting cell growth and Darpp-32 in inhibiting cell growth. Decreased tumor growth in *Ppp1r1b* knockout mice also suggests that t-Darpp plays a direct role, predominant to Darpp-32, in mammary tumor development. These results indicate that transgenic mouse mammary tumor models might be valuable tools for future investigation of Darpp-32 and t-Darpp in breast cancer.

**Electronic supplementary material:**

The online version of this article (doi:10.1186/1476-4598-13-192) contains supplementary material, which is available to authorized users.

## Background

Darpp-32 (dopamine and cAMP regulated phosphoprotein of 32 kD) is well known for its primary role as an integrator and regulator of neurotransmission [1]. It has also recently been linked to cancer. El-Rifai and colleagues were the first to find Darpp-32 and a truncated variant, t-Darpp, in gastric carcinoma patient samples [2]. Darpp-32 and t-Darpp expression has since been identified in breast cancer as well as numerous other types of malignancies [3].

The function of Darpp-32 in non-neuronal cells and in cancer is not well understood. The majority of data suggests that it has a growth inhibitory effect and a potentially antagonistic relationship with t-Darpp [4–8]. The truncated t-Darpp protein lacks 36 amino acids at the N-terminus, including a phosphorylation site critical to the function of Darpp-32 as a phosphatase inhibitor [2, 9, 10]. t-Darpp’s mechanism of action is not known, but it has reported activity for increasing cell growth, inhibiting apoptosis, and promoting drug resistance in cancer cells [11–17].

Darpp-32 and t-Darpp are expressed from the gene *PPP1R1B* (protein phosphatase 1, regulatory (inhibitor) subunit 1B), utilizing unique transcription and translation start sites for their respective mRNAs [10]. In the human breast, Darpp-32 is expressed in both normal and malignant tissue, whereas t-Darpp is typically found only in breast adenocarcinomas [3, 11, 16]. The functional significance of Darpp-32 and t-Darpp expression in breast cancer is not known, but the aberrant expression of these proteins in cancer, as compared to the relatively low levels observed in healthy tissue, indicates a possible role for these proteins in tumor growth and progression.

To date, the role of Darpp-32 and t-Darpp in cancer has been studied only in patient samples, cell lines, and xenograft mouse models. Transgenic mouse tumor models such as MMTV-Neu and MMTV-PyMT [18], in which mice develop spontaneous mammary tumors, have heretofore been unsuitable for the investigation of Darpp-32 and t-Darpp due to the lack of published data on these proteins in mouse mammary tissue. The existence of a truncated form of Darpp-32 has not been reported for any cell type in mice, although t-Darpp expression has been noted in the rat brain [19]. The purpose of this study was to determine if Darpp-32 and t-Darpp are expressed in mouse mammary tissue and to see if their expression patterns are similar between humans and mice. Using a *Ppp1r1b* knockout mouse, we also sought to determine the role of Darpp-32 and t-Darpp in breast tumorigenesis and progression.

## Results

### Darpp-32, but not t-Darpp, is expressed in normal mouse mammary tissue

**Figure 1:**
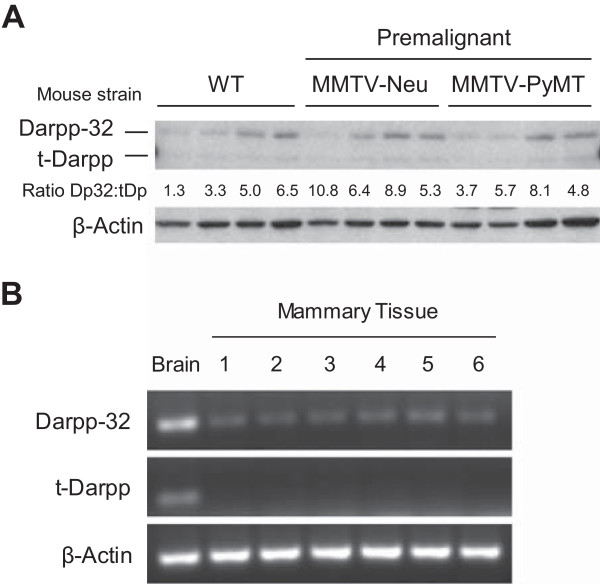
**Darpp-32 and t-Darpp expression in normal mouse mammary tissue. (A)** Darpp-32 and t-Darpp protein levels were measured by Western analysis. β–Actin levels were measured as a loading control. Abdominal and inguinal mammary pads (#4/5 and #9/10) were collected from wild-type FVB mice and from premalignant transgenic mammary tumor mice. Mammary cells from two mice per strain (two fat pads per mouse) were analyzed. The ratio of Darpp-32 to t-Darpp (Dp32:tDp) protein expression was calculated for each sample. **(B)** Darpp-32 and t-Darpp RNA levels were measured by traditional RT-PCR using isoform-specific primers. β–Actin levels were measured as a loading control. RNA from a wild-type mouse brain was used as a positive control. Mammary tissue samples were: 1) NMuMG mouse mammary epithelial cell line, 2–5) four separate wild-type mammary pads (non-dissociated) from two mice and 6) dissociated wild-type mammary cells pooled from two mice.

In humans, the full-length transcript encoding Darpp-32 is sometimes detected in normal, healthy epithelial tissue. t-Darpp, on the other hand, is rarely expressed [3]. To determine the expression patterns in normal mouse mammary tissue, we collected multiple mammary pads from wild-type mice and from pre-malignant MMTV-Neu and MMTV-PyMT transgenic mice. Because mouse mammary pads are composed mostly of adipose tissue, the collected tissue was enzymatically dissociated to isolate mammary cells. Darpp-32 and t-Darpp protein (Figure 1A) and mRNA (Figure 1B) levels were examined, using mouse brain as a positive control. The majority (67%) of normal mammary samples expressed Darpp-32 protein, whereas none expressed detectable levels of t-Darpp (Figure 1A). Similar results were observed at the mRNA level. All normal mammary samples, including both dissociated and whole mammary pads as well as the normal mouse mammary epithelial cell line NMuMG, expressed Darpp-32 mRNA but none expressed t-Darpp mRNA (Figure 1B).

### Darpp-32 and t-Darpp are expressed in mouse mammary tumors

**Figure 2:**
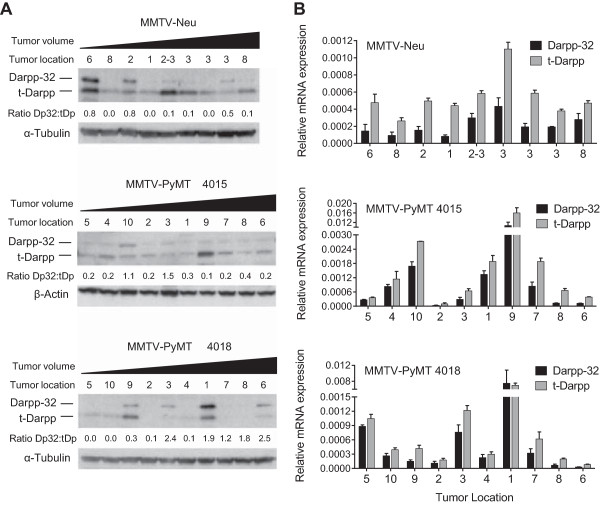
**Darpp-32 and t-Darpp expression in transgenic mouse mammary tumors.** Nine individual tumors were collected from nine different MMTV-Neu mice. All ten MMTV-PyMT tumors were collected from two mice. Samples are arranged from smallest to largest (tumor volume) and the tumor location refers to the mammary pad from which the tumor originated. **(A)** Darpp-32 and t-Darpp protein levels were measured by Western analysis. α–Tubulin and β–Actin were used as loading controls. The ratio of Darpp-32 to t-Darpp (Dp32:tDp) protein expression was calculated for each sample. **(B)** Darpp-32 and t-Darpp RNA levels were measured by SYBR Green quantitative real-time RT-PCR using isoform-specific primers. Data was normalized to β–Actin; mean ± standard deviation.

**Figure 3:**
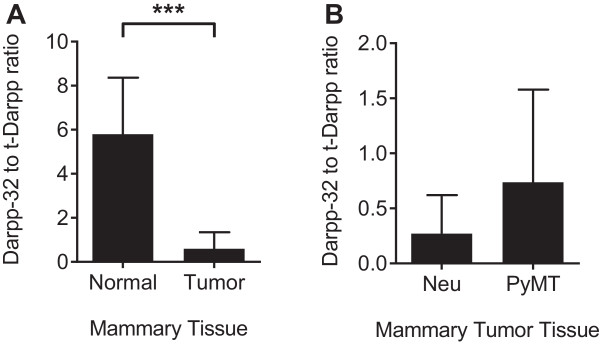
**Differential ratio of Darpp-32 to t-Darpp in normal and tumor tissue.** Darpp-32 and t-Darpp protein levels, from the Figures 1A and 2A Westerns, were quantified using ImageJ software and expressed as the ratio of Darpp-32 to t-Darpp. The ratio was calculated for **(A)** normal (*n* = 12) and all malignant (*n* = 29) mouse mammary tissue (MMTV-Neu and MMTV-PyMT combined) and **(B)** MMTV-Neu (*n* = 9) versus MMTV-PyMT (*n* = 20) tumor tissue. Shown are means ± standard deviation; *** *p* < 0.0001.

Both Darpp-32 and t-Darpp are overexpressed in human breast cancers [3, 11]. To determine if this is the case in mouse mammary tumors, we used two different transgenic models of mouse breast cancer, MMTV-Neu and MMTV-PyMT. Tumors of different sizes and from different mammary pads were collected from both models. Single tumors were collected from nine MMTV-Neu mice whereas multiple tumors were collected from two different MMTV-PyMT mice. Protein and mRNA were collected from each tumor and analyzed for Darpp-32 and t-Darpp expression. In contrast to its expression in the majority of normal mammary tissue samples, Darpp-32 protein was seen in only 24% of mouse mammary tumors. Conversely, t-Darpp, which was not detected in normal tissue, was expressed at significant levels in 52% of mammary tumors. There did not seem to be a correlation between Darpp-32 or t-Darpp expression and mouse model, tumor size or tumor location (Figure 2A). Corresponding results were observed at the mRNA level, with detectable levels of t-Darpp mRNA in the vast majority of tumors (Figure 2B). The ratio of Darpp-32 to t-Darpp protein was significantly lower in the tumor samples than in the normal mammary tissue (*p* < 0.0001, Figure 3A). No significant difference in the protein ratios was observed between tumor models (Figure 3B). These results suggest a shift in protein levels from exclusively Darpp-32 to predominantly t-Darpp during mouse mammary malignant transformation, reminiscent of the expression patterns observed in human breast tissue.

### Knockout of Darpp-32 and t-Darpp leads to decreased growth of spontaneous mouse mammary tumors

**Figure 4:**
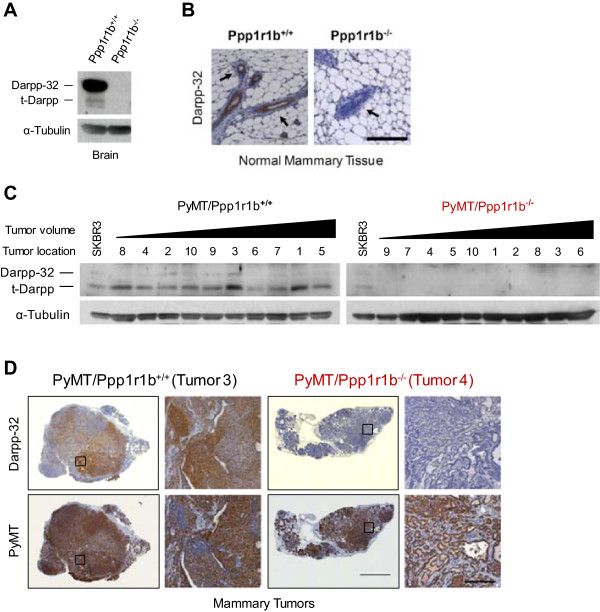
**Darpp-32 and t-Darpp expression in** ***Ppp1r1b*** **knockout mice. (A)** Western analysis of Darpp-32 and t-Darpp protein levels in brain tissue from wild-type (Ppp1r1b^+/+^) and knockout (Ppp1r1b^-/-^) mice. α–Tubulin was used as a loading control. **(B)** Mammary pads (abdominal and inguinal, #4/5) were collected from 20-week-old Ppp1r1b^+/+^ and Ppp1r1b^-/-^ mice. Formalin-fixed sections were stained for Darpp-32 (5× magnification, scale bar = 200 μm). Black arrows indicate mammary epithelial cells surrounded by the adipose tissue of the mammary fat pad. **(C)** Western analysis of Darpp-32 and t-Darpp protein levels in mammary tumor tissue. α–Tubulin was used as a loading control. All ten tumors were collected from 20-week-old wild-type (PyMT/Ppp1r1b^+/+^) and knockout (PyMT/Ppp1r1b^-/-^) mice carrying the MMTV-PyMT transgene. Tumor samples are arranged from smallest to largest (tumor volume) and the tumor location is specified. SKBR3 human breast cancer cells were used as a reference for Darpp-32 and t-Darpp expression. **(D)** Mammary tumor samples were formalin-fixed and serial sections were stained for Darpp-32 and PyMT (5× magnification in a 6 × 6 tile, scale bar = 2000 μm; images enlarged from the boxed regions are at 10× magnification, scale bar = 200 μm). Tumor numbers correspond to the numbering in panel **C**.

The previous experiments show that Darpp-32 and t-Darpp are differentially expressed in normal and malignant mouse mammary tissue. To determine if these proteins have a direct effect on tumorigenesis and growth, we crossed *Ppp1r1b* knockout mice with the MMTV-PyMT mammary tumor model and examined the rate of tumor initiation, growth and metastasis in the wild-type (PyMT/Ppp1r1b^+/+^) and knockout (PyMT/Ppp1r1b^-/-^) *Ppp1r1b* backgrounds. The way in which the *Ppp1r1b* knockout mouse was engineered, with a neo cassette inserted into the translational start site of Darpp-32 [20], led to the knockout of both Darpp-32 and t-Darpp expression in these mice. No Darpp-32 or t-Darpp protein was detected in normal tissue from Ppp1r1b^-/-^ mice (*Ppp1r1b* knockout in the absence of PyMT, Figure 4A-B), nor tumor tissue from PyMT/Ppp1r1b^-/-^ mice (Figure 4C-D).

**Figure 5:**
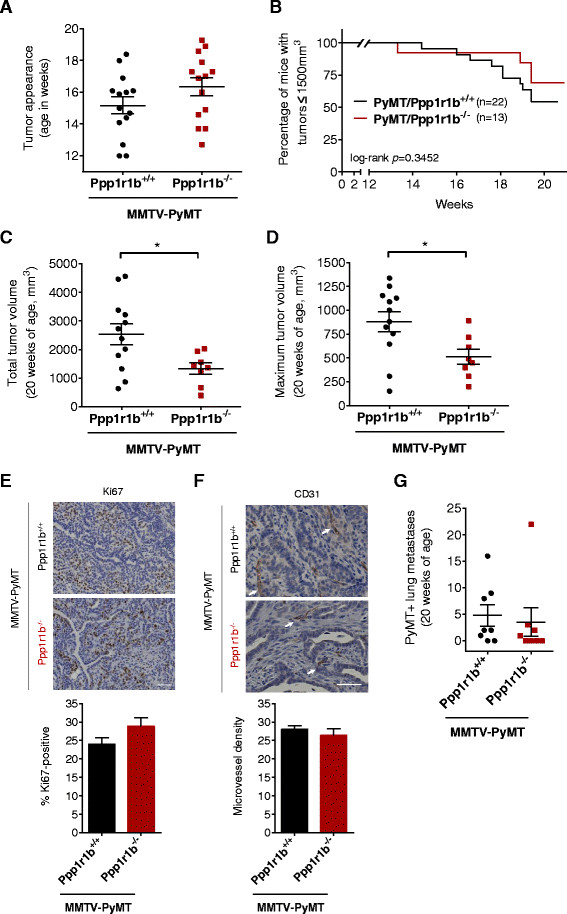
**Tumor growth in PyMT/Ppp1r1b** ^**+/+**^ **and PyMT/Ppp1r1b** ^**-/-**^ **mice. (A)** The age at which tumors first appeared (tumor volume 100–300 mm^3^) was recorded for wild-type (PyMT/Ppp1r1b^+/+^) and knockout (PyMT/Ppp1r1b^-/-^) tumor mice *(n* = 14 per group). Individual ages and the mean age at appearance (±standard error of the mean) are shown. **(B)** Kaplan–Meier survival plot showing the age at which mice were euthanized because of extensive tumor burden (maximum tumor volume >1500 mm^3^). **(C-D)** Mice surviving to 20 weeks of age were euthanized and tumor volume was measured. **(C)** Total tumor volume in these mice was calculated as the sum of the individual tumor volumes for each mouse. **(D)** Maximum tumor volume was defined as the volume of the largest individual tumor per mouse (*n* = 8–12 per group); mean ± standard error of the mean, *p < 0.05. **(E-F)** Tumors were collected at 20 weeks of age, formalin-fixed, sectioned and immuno-stained for Ki67 and CD31. **(E)** Representative tumor sections and bar graph showing the mean (± standard error of the mean) number of Ki67-positive cells per field in 10 nonoverlapping fields from three tumors per group (25× magnification, scale bar = 50 μm). **(F)** Representative tumor sections and bar graph showing the mean (± standard error of the mean) microvessel density determined by counting the number of CD31-positive endothelial clusters (white arrows) in 10 nonoverlapping fields from three tumors per group (25× magnification, scale bar = 50 μm). **(G)** Number of PyMT-positive metastatic nodules in each lung analyzed at 20 weeks of age. Individual lung counts (*n* = 8 per group) and the mean (± standard error of the mean) for each genotype group are shown.

To determine if the absence of Darpp-32 and t-Darpp affected tumor growth, we monitored mice weekly from 8 to 20 weeks of age, both for the emergence of tumors and for tumor volume. Control Ppp1r1b^-/-^ mice did not develop tumors in this timeframe, whereas tumors began appearing in PyMT/Ppp1r1b^-/-^ mice as early as 13 weeks. PyMT/Ppp1r1b^-/-^ mice did not show either significantly earlier or delayed tumor formation relative to PyMT/Ppp1r1b^+/+^ mice (Figure 5A). Similarly, the percentage of mice that needed to be sacrificed early due to extensive tumor burden (tumor volume >1500 mm^3^) was not changed by the absence of Darpp-32 and t-Darpp (Figure 5B). A significant reduction in tumor volume, both in terms of total tumor volume (*p* = 0.023, Figure 5C) and the volume of the largest tumor per mouse (*p* = 0.021, Figure 5D), was observed in PyMT/Ppp1r1b^-/-^ mice at 20 weeks, compared to PyMT/Ppp1r1b^+/+^ mice at the same time point. We measured nuclear Ki67 expression in tumors as another marker of proliferation and found no difference in Ki67 staining between PyMT/Ppp1r1b^+/+^ and PyMT/Ppp1r1b^-/-^ mice (Figure 5E).

MMTV-PyMT is well known as a murine model of tumor metastasis, with the majority of tumor bearing mice developing metastasis to the lung [21]. Because Darpp-32 has been shown to inhibit breast cancer cell migration [5, 6], we examined the possible effects of Darpp-32 or t-Darpp on early tumor progression. Vascularization is one of the first steps in the metastatic cascade, with increased blood vessel formation facilitating the dissemination of primary tumor cells throughout the body [22]. We therefore examined expression of the vascular endothelial marker CD31 in tumors. We observed no significant difference in the number of CD31-positive endothelial cell clusters between PyMT/Ppp1r1b^+/+^ and PyMT/Ppp1r1b^-/-^ mice (Figure 5F).

To investigate possible effects on metastasis, we collected lungs from 20-week-old tumor bearing mice and looked for PyMT-positive metastatic nodules (Additional file 1: Figure S1A). PyMT/Ppp1r1b^-/-^ mice did not have a statistically different number or size of metastases compared with PyMT/Ppp1r1b^+/+^ mice, although there might have been a pattern of fewer metastatic lung nodules in the knockout mice (Figure 5G and Additional file 1: Figure S1B). We also looked at Darpp-32 and t-Darpp expression in PyMT/Ppp1r1b^+/+^ lung metastases. Since t-Darpp is a truncated version of Darpp-32, there are no antibodies that will detect just t-Darpp (and not Darpp-32) by immunohistochemistry. Instead, we used an antibody that recognizes just Darpp-32 and another that recognizes both Darpp-32 and t-Darpp, and resulting staining patterns were examined by a veterinary pathologist. We saw no clear patterns in the wild-type metastases to suggest under- or over-expression of Darpp-32 or t-Darpp in metastases (data not shown). Taken together, these results suggest that Darpp-32 and t-Darpp are not directly involved in metastasis in this mouse model (also see the Discussion).

## Discussion

This is the first time that Darpp-32 and t-Darpp have been examined in mouse mammary tissue and the first evidence of t-Darpp expression in mice. Of greater interest is the observation that expression seems to shift from a predominance of Darpp-32 in normal mouse mammary tissue towards a predominance of t-Darpp in malignant mammary tissue. This suggests that t-Darpp by itself or the ratio of Darpp-32 to t-Darpp might be significant in determining a cell’s tumorigenicity. We have previously reported that Darpp-32 and t-Darpp appear to have opposing functions in breast cancer cell lines, with t-Darpp promoting cell survival and drug resistance and Darpp-32 being growth inhibitory and reversing t-Darpp’s effect [15]. This is consistent with the idea that it is not just the overexpression of one protein or the other, but rather their relative expression levels that determine an overall growth or survival phenotype. This might be a general phenomenon of normal versus malignant tissue rather than a dose–response phenomenon, since we did not see a direct correlation between the ratio of Darpp-32:t-Darpp and tumor size.

We also saw variable expression of each protein from tumor to tumor, similar to what is observed in humans [3]. Expression did not seem to correlate with tumor location or size. This could be a product of tumor heterogeneity or some underlying yet unknown factor that determines whether Darpp-32 or t-Darpp are expressed. There is little to no information on the transcriptional regulation of Darpp-32 and t-Darpp. It will be interesting to determine what regulates expression from one promoter versus the other, perhaps as an initiating event in tumorigenesis when transcription from the downstream t-Darpp promoter is pronounced.

One possibility, given the presence of a CpG island within the first exon of Darpp-32, is that Darpp-32 is down-regulated by tumor-specific hypermethylation, thus permitting transcription from the downstream t-Darpp promoter. Such expression would be expected to promote growth and cell survival, thus perhaps contributing to tumorigenesis. Consistent with this idea, we have data from human breast cell lines indicating that the Darpp-32 promoter is in fact subject to silencing through hypermethylation in malignant versus non-malignant cells (unpublished observations). The shift in expression from an absence of t-Darpp in healthy tissue toward a predominance of t-Darpp in tumor tissue further supports this theory. That Darpp-32 is sometimes co-expressed with t-Darpp in malignant tissue suggests that silencing is incomplete or that DNA methylation is only one of several mechanisms responsible for regulating gene expression.

Because Darpp-32 and t-Darpp were jointly knocked out in this mouse model, interpretation of the data is somewhat difficult. Nevertheless, our results with *Ppp1r1b* knockout mice are consistent with a role for t-Darpp in promoting tumor growth. We observed lower overall tumor volume in PyMT/Ppp1r1b^-/-^ mice at 20 weeks of age and trends toward delayed tumor appearance in these mice as well. One explanation could be that t-Darpp plays a role predominant to Darpp-32 in mammary tumor development. In normal mammary tissue, Darpp-32 might have a type of tumor suppressor effect, whereas the decrease in Darpp-32 and gain of t-Darpp expression during tumorigenesis would tend to promote tumor growth. Some PyMT tumors did retain Darpp-32 expression, albeit at low levels, and this expression was almost always accompanied by t-Darpp expression. This might offset any tumor suppressor effects that Darpp-32 has in tumor tissue. Indeed if Darpp-32 and t-Darpp have antagonistic effects on malignant cell growth, as we and others have suggested [4–8, 15], then knocking out both proteins could potentially have a net neutral effect on tumor growth. This might explain the lack of effect on steady-state Ki67 levels and the modest effects on tumor appearance and volume that we observed. It will be interesting to see how individual knockout of either Darpp-32 or t-Darpp affects tumor development in either sporadic or PyMT-driven tumorigenesis.

Another complicating factor originates with the MMTV-PyMT tumor model used for these knockout experiments. PyMT is a strong driver of cell proliferation and tumors develop very quickly in 100% of mice. We observed a median age of tumor initiation in PyMT/Ppp1r1b^+/+^ mice of around 15 weeks, with some mice reaching maximum tumor volume as early as 1–2 weeks later. This might make it difficult to detect changes in tumorigenesis or cell proliferation after knockout of a possible tumor suppressor (Darpp-32) or another tumor-promoting protein (t-Darpp) in the PyMT background. Moreover, rapid tumor development in MMTV-PyMT mice does not allow much time for vascular and metastatic development before mice reach maximum tumor burden, thus complicating the investigation of these stages in tumor progression. Looking at the effects of *Ppp1r1b* knockout in a slower growing tumor model, such as the MMTV-Neu model [23, 24], might be helpful in elucidating the specific role of *Ppp1r1b* in both tumorigenesis and the processes associated with metastasis.

In addition, larger sample sizes are likely needed to gain a complete understanding of the role of *Ppp1r1b* in metastasis. With only 80–94% of PyMT/Ppp1r1b^+/+^ mice developing metastases [21], the sample size of eight mice per group was likely too small to recognize anything but very large differences in metastasis between PyMT/Ppp1r1b^+/+^ and PyMT/Ppp1r1b^-/-^ mice. It is perhaps notable that a single PyMT/Ppp1r1b^-/-^ female, with a total of 22 lung metastases, inflated the PyMT/Ppp1r1b^-/-^ group average from 0.86 to 3.50 metastatic nodules per mouse, thus skewing the metastasis data in this group of mice considerably (Figure 5G). However, this outlier had little effect on the statistical significance of the data given the variability in metastases observed within each group. Interestingly, this same mouse also had the largest total tumor volume among the PyMT/Ppp1r1b^-/-^ cohort. Larger tumor volume did seem to be roughly associated with a higher number of lung metastases (see Additional file 1: Figure S1C), so it is not surprising that PyMT/Ppp1r1b^-/-^ mice, with smaller primary tumors, on average, would also have fewer lung metastases than PyMT/Ppp1r1b^+/+^ mice. Taken together, our data suggest a more important role for *Ppp1r1b* in affecting tumor growth than metastasis per se, with t-Darpp apparently having the predominant role to play. Others have suggested that Darpp-32 acts to inhibit metastasis [5, 6], but again we might have missed such an effect with the current model system in which both Darpp-32 and t-Darpp are simultaneously knocked out.

## Conclusions

The shift from Darpp-32 to t-Darpp during mouse mammary tumorigenesis is reminiscent of the expression patterns observed in humans and is consistent with the theorized opposing functions of t-Darpp and Darpp-32 in promoting and inhibiting tumor progression, respectively. The data from the *Ppp1r1b* knockout tumor mice suggest that the *Ppp1r1b* gene products, t-Darpp in particular, could have a direct role in mammary tumor development in MMTV-PyMT mice. Further investigation is needed, but it seems as though mouse mammary tumor models could be a useful tool to better understand the expression patterns and regulation of Darpp-32 and t-Darpp in breast cancer.

## Methods

### Mice

Wild-type mice were provided by the City of Hope Animal Resources core facility. MMTV-Neu (strain FVB/N-Tg(MMTVneu)202Mul/J) [23, 24] and MMTV-PyMT (strain FVB/N-Tg(MMTV-PyVT)634Mul/J) [21, 25] mice were obtained from Jackson Laboratory (Bar Harbor, ME). Darpp-32 (*Ppp1r1b*) knockout mice were kindly provided by Paul Greengard (The Rockefeller University, New York, NY) [20]. Mouse breeding, monitoring for tumor formation, and tissue collection and analysis are described below. Mice were sacrificed for tissue and tumor collection by CO_2_ inhalation. Experimental protocols were approved by the City of Hope Institutional Animal Care and Use Committee (IACUC).

### Cell culture

The mouse mammary epithelial cell line NMuMG was kindly provided by Emily Wang (City of Hope, Duarte, CA). NMuMG cells were maintained in DMEM with 10% FBS and 1% penicillin/streptomycin in 5% CO_2_. The SKBR3 human breast cancer cell line was obtained from the American Type Culture Collection (Rockville, MD). SKBR3 cells were maintained in McCoy’s Medium 5A with 10% FBS, 1% penicillin/streptomycin, and 1% L-glutamine in 5% CO_2_.

### Tissue collection

Brain tissue from sacrificed mice was extracted and frozen in liquid nitrogen. Normal mammary pads (abdominal and inguinal, #4/5 and #9/10) were resected and either frozen whole in liquid nitrogen or immediately dissociated for mammary cell isolation. Dissociation was conducted using a Collagenase/Hyaluronidase dissociation solution from Stem Cell Technologies (Vancouver, BC, Canada) according to the manufacturer’s instructions. Briefly, mammary pads were minced and incubated in the dissociation solution at 37°C for 2 hours with frequent vortexing. After centrifugation at 350 × *g* for 5 minutes, the supernatant containing the liquefied fat tissue was discarded and the mammary cells, enriched for epithelial cell organoids as well as stromal cells and lymphocytes, were washed in preparation for cell lysis and RNA isolation. For collection of tumors, tumor length (L) and width (W) were measured post-mortem (before dissection) using an electronic caliper. Tumor volume (V) was calculated using the formula V = ½ (L × W^2^) [26, 27]. Tumors were resected and immediately frozen in liquid nitrogen. Frozen tissue was cut on dry ice and homogenized in preparation for the isolation of cell lysates and RNA.

### Western analysis

Mouse tissue and cultured cell lysates were collected on ice in RIPA buffer from Thermo Scientific (Waltham, MA) supplemented with 1× protease inhibitor cocktail from Roche Applied Science (Indianapolis, IN). Protein concentration was determined by RC DC protein assay purchased from Bio-Rad Laboratories (Hercules, CA). 30 μg of protein from each sample was loaded onto a 12% SDS-PAGE gel for protein separation and proteins were transferred to a nitrocellulose membrane. 5% non-fat dry milk was used for a blocking buffer and for primary antibody incubation. Primary antibodies included: an antibody that recognizes both Darpp-32 and t-Darpp (#H62) from Santa Cruz Biotechnology (Santa Cruz, CA) and antibodies to α-Tubulin (#T5168) and β-Actin (#A4700) from Sigma-Aldrich Corporation (St. Louis, MO). Secondary antibodies were horseradish peroxidase-conjugated anti-mouse IgG and anti-rabbit IgG antibodies from Cell Signaling Technology (Danvers, Massachusetts). Secondary antibody was detected using an ECL Plus kit from Thermo Fisher Scientific. Protein expression was quantified using ImageJ software and expressed as relative density, normalized to loading control values. Mammary tissue was arbitrarily considered positive for protein expression when the relative density was greater than 0.5.

### RNA preparation and RT-PCR

Total RNA was isolated and purified using the Qiagen RNeasy kit (Valencia, CA). Residual DNA was removed from samples using the Ambion® TURBO DNA-*free*™ kit from Life Technologies (Carlsbad, CA). RNA was reverse transcribed to cDNA using random primers and SuperScript III Reverse Transcriptase from Life Technologies. Darpp-32 and t-Darpp mRNA levels were analyzed using either traditional or quantitative RT-PCR. Traditional PCR (1 cycle of 30 sec at 98°C, 30 cycles of 30 sec at 98°C, 30 sec at 58°C, 15 sec at 72°C, and 5 min incubation at 72°C) was performed using Finnzyme Phusion Hot Start II DNA Polymerase from Thermo Scientific. Quantitative RT-PCR (1 cycle of 3 min at 95°C, 40 cycles of 10 sec at 95°C, 30 sec at 58°C, and a melting curve 55–95°C) was performed using the PerfeCTa® SYBR® Green SuperMix from Quanta BioSciences (Gaithersburg, MD). Quadruplicate measurements were made on a single isolation of RNA from each sample analyzed. Primers 5′-AGATTCAGTTCTCTGTGCCCG-3′ and 5′-GGTTCTCTGATGTGGAGAGGC-3′ were used to amplify Darpp-32 mRNA; primers 5′-CGATGGTGAGGTGCCCCTAT-3′ and 5′-CTCCTCTGGTGAGGAGTGCT-3′ were used to amplify t-Darpp mRNA; and primers 5′-AGATCAAGATCATTGCTCCTCCC-3′ and 5′-AAGGGTGTAAAACGCAGCTC-3′ were used to amplify β-Actin mRNA.

### *In vivo*tumorigenesis

*Ppp1r1b* knockout mice with a pure C57BL/6 background were crossed with MMTV-PyMT mice with a 50:50 mixed C57BL/6 and FVB background. To ensure that all experimental mice contained the same background percentages of C57BL/6 and FVB (75:25, respectively), F1 mice were back-crossed for two generations and genotyped at each generation by PCR analysis of tail DNA. Ppp1r1b^+/+^, Ppp1r1b^-/-^, PyMT/Ppp1r1b^+/+^ and PyMT/Ppp1r1b^-/-^ mice from the F3+ generation(s) were used in all reported experiments. Starting at 8 weeks of age, mice were monitored weekly for the appearance of palpable tumors. A palpable tumor was defined as a tumor with a volume between 100–300 mm^3^. Tumor volume was measured weekly until mice were sacrificed at 20 weeks of age. Mice were sacrificed prior to 20 weeks of age when a tumor reached a volume >1500 mm^3^ (in accordance with the approved IACUC protocol’s definition of excessive tumor burden), and these mice were not included in any of the 20-week data sets. A final measure of tumor volume was taken post-mortem before tissue was collected for molecular and pathological analysis. Total tumor volume was calculated for each mouse as the sum of the individual tumor volumes. Maximum tumor volume was defined as the volume of the largest individual tumor in each mouse.

### Histology and immunohistochemistry

Lungs obtained from sacrificed mice were perfused intra-tracheally and fixed in 10% buffered formalin. Tumors and normal mammary pads (abdominal and inguinal, #4/5) were resected and fixed in 10% buffered formalin. Tissues were paraffin embedded and sectioned (5 μm thick). Serial sections were stained with hematoxylin and eosin (H&E) and prepared for immunohistochemical analysis. Slides were deparaffinized in xylene followed by 100–70% ethanols, quenched in 3% hydrogen peroxide and pretreated to promote antigen retrieval with either a High pH or DIVA buffer. Slides were then pretreated with a blocking serum and incubated in primary antibody. Primary antibodies were: a rabbit monoclonal antibody specific for Darpp-32 (#40801) from Abcam (Cambridge, MA) at 1:500 dilution in PBS for 30 min at room temperature (RT); a rat monoclonal antibody specific for Polyoma virus medium T antigen (PyMT, #NB-100-2749) from Novus Biologicals LLC (Littleton, CO) at 1:300 dilution in PBS for 30 min at RT; a goat polyclonal antibody specific for CD31/PECAM1 (#sc1506) from Santa Cruz Biotechnology at 1:500 dilution in PBS for 30 min at RT; and a rabbit polyclonal antibody specific for Ki67 (#PA5-19462) from Thermo Scientific at 1:200 dilution in PBS for 30 min at RT. Secondary antibodies were biotinylated anti-rabbit (#BA-1000), anti-goat (#BA-9500) and anti-rat (#BA-4001) from Vector Lab (Burlingame, CA), and an anti-rabbit secondary polymer from Dako (#K4003, Carpinteria, CA). Slides were then incubated with the chromogen diaminobenzidine tetrahydrochloride, counterstained with hematoxylin and mounted with a permanent mounting media.

The histological and immunohistochemical results were visually inspected by a veterinary pathologist while blinded to the genotype of each specimen. All images were taken using a Zeiss Axio Observer Z1 Inverted microscope with a Hamamatsu EMCCD C9100-13 Monochromo camera and Zeiss AxioVision 4.8 software. Tumors and mammary pads were imaged using either the 5×/0.16NA Phan-NeoFluar Phase objective (5× magnification in a 6 × 6 tile on an automated stage) or the 10×/0.5NA Fluar DIC objective (10× magnification). Ki67 and CD31 expression in mammary tumors was imaged using the 25×/0.8NA LCI PlanApo Multi Immersion DIC, Correction Collar, LD objective (25× magnification). The number of Ki67-positive cells was counted in 10 nonoverlapping fields in three tumors per group [28]. Microvessel density was quantified by counting the number of CD31-positive endothelial clusters in 10 nonoverlapping fields in three tumors per group (25× magnification) [28, 29].

### Lung metastasis

Lung sections stained with H&E (three sagittal sections separated by 15 μm, cut from deep within each lung) were visually examined for lung metastasis by a veterinary pathologist. Lung sections stained for PyMT expression were scanned using a Zeiss Axio Observer Z1 Inverted microscope with a Hamamatsu EMCCD C9100-13 Monochromo camera and 5×/0.16NA Phan-NeoFluar Phase objective using Zeiss AxioVision 4.8 software (5× magnification in a 5 × 10 tile on an automated stage). Image Pro Premier 9.0 imaging software was used to count the total number of PyMT-positive metastatic lung nodules per mouse and to measure the area of each nodule to determine their classification as either a micrometastasis (≤0.025 mm^2^) or a macrometastasis (>0.025 mm^2^).

### Statistical analysis

Statistically significant differences were calculated using the GraphPad Prism 6.0 statistical program. Differences between groups were determined by the two-tailed Student’s *t*-test, and Kaplan-Meier plots of survival were analyzed using the log-rank Mantel Cox test. *p* values <0.05 were considered significant.

## Electronic supplementary material

Additional file 1: Figure S1: Lung metastasis. Lung tissue was collected at 20 weeks of age and examined for metastasis. **(A)** Formalin-fixed lungs were sectioned and stained with H&E and for PyMT expression (5× magnification in a 4 × 9 tile, scale bar = 2000 μm). The boxed regions are shown as enlarged images in the bottom panels (5× magnification in a 2 × 3 tile, scale bar = 500 μm). These highlight the differences between micrometastases (white arrows, ≤0.025 mm^2^) and macrometastases (black arrows, >0.25 mm^2^). **(B)** The number of PyMT-positive micro- and macrometastases in each lung were counted (*n* = 8 per group); mean ± standard error of the mean. **(C)** The relationship between tumor volume and metastases, including the Pearson correlation coefficient and best fit linear regression. (PDF 122 KB)

## Abbreviations

Darpp-32: dopamine and cAMP regulated phosphoprotein 32 kD
t-Darpp: truncated Darpp-32
*PPP1R1B*: Protein phosphatase 1, regulatory (inhibitor) subunit 1B
PyMT: Polyoma virus middle T antigen
hematoxylin and eosin: H&E.
