# Supplementary figures and images for: Darpp-32 and t-Darpp are differentially expressed in normal and malignant mouse mammary tissue
Source: Mol Cancer. 2014 Aug 15;13:192. doi: 10.1186/1476-4598-13-192 (PMC4147176; doi:10.1186/1476-4598-13-192)

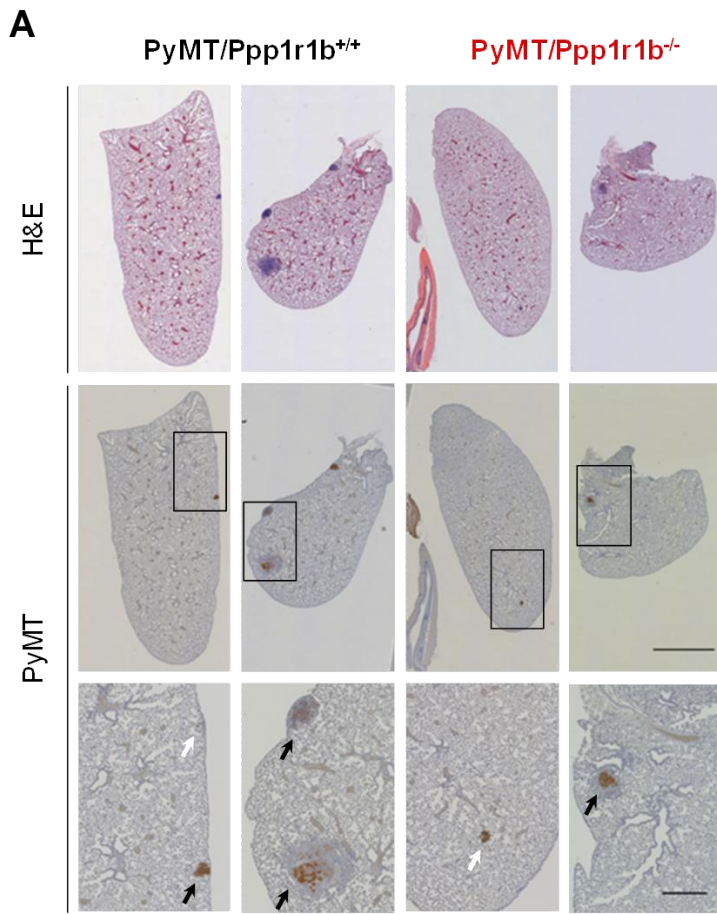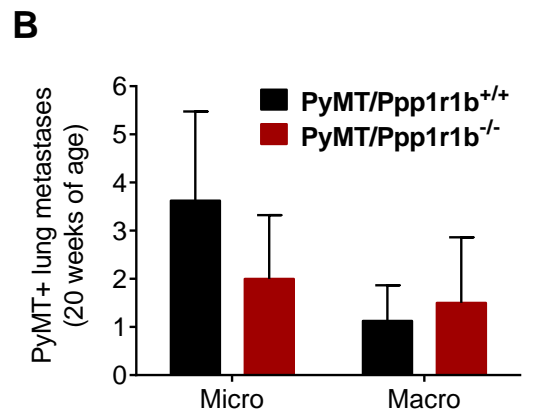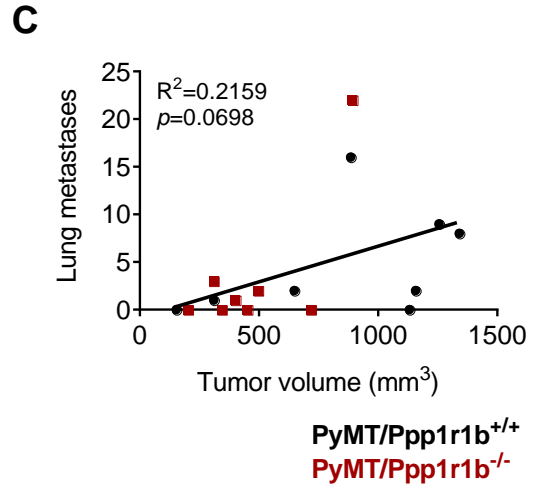

Supplement: Supplementary file 1 — Additional file 1: Figure S1: Lung metastasis. Lung tissue was collected at 20 weeks of age and examined for metastasis. (A) Formalin-fixed lungs were sectioned and stained with H&E and for PyMT expression (5× magnification in a 4 × 9 tile, scale bar = 2000 μm). The boxed regions are shown as enlarged images in the bottom panels (5× magnification in a 2 × 3 tile, scale bar = 500 μm). These highlight the differences between micrometastases (white arrows, ≤0.025 mm2) and macrometastases (black arrows, >0.25 mm2). (B) The number of PyMT-positive micro- and macrometastases in each lung were counted (n = 8 per group); mean ± standard error of the mean. (C) The relationship between tumor volume and metastases, including the Pearson correlation coefficient and best fit linear regression. (PDF 122 KB) [file 12943_2014_1394_MOESM1_ESM.pdf]
